# Predicting the bounds of large chaotic systems using low-dimensional manifolds

**DOI:** 10.1371/journal.pone.0179507

**Published:** 2017-06-23

**Authors:** Asger M. Haugaard

**Affiliations:** University of Oxford, Medical sciences division, Oxford, OX3 9DU, United Kingdom; Tongji University, CHINA

## Abstract

Predicting extrema of chaotic systems in high-dimensional phase space remains a challenge. Methods, which give extrema that are valid in the long term, have thus far been restricted to models of only a few variables. Here, a method is presented which treats extrema of chaotic systems as belonging to discretised manifolds of low dimension (low-D) embedded in high-dimensional (high-D) phase space. As a central feature, the method exploits that strange attractor dimension is generally much smaller than parent system phase space dimension. This is important, since the computational cost associated with discretised manifolds depends exponentially on their dimension. Thus, systems that would otherwise be associated with tremendous computational challenges, can be tackled on a laptop. As a test, bounding manifolds are calculated for high-D modifications of the canonical Duffing system. Parameters can be set such that the bounding manifold displays harmonic behaviour even if the underlying system is chaotic. Thus, solving for one post-transient forcing cycle of the bounding manifold predicts the extrema of the underlying chaotic problem indefinitely.

## Introduction

Coupled systems of non-linear ordinary differential equations (ODEs) are ubiquitous in scientific literature and appear as the end result of efforts to mathematically model phenomena from practically every branch of science. The desire for quantitative accuracy often leads to such models containing large numbers of variables, for example, as a consequence of a highly resolved finite element mesh, an in-silico model of human tissue—be it heart, tumour or brain—containing many simulated cells, or an electrical circuit with many nodes. Systems of ordinary differential equations with non-linear terms have the potential to display chaotic behaviour, in which case their solutions evolve on “strange” attractors of fractal dimension.

The ability to precisely predict the extrema of large chaotic systems has obvious utility across many fields. While bifurcation analysis serves to characterise the qualitative behaviour of a system, extrema prediction adds quantitative knowledge. Perhaps the clearest benefit is where state variables have critical values, e.g., in predator-prey systems where reaching a critical value might spell extinction of a species or in neuron- or cardiac myocyte models, where reaching certain states might initiate seizures or arrhythmias. Even when such dichotomous phenomena are not in play, extrema may still be important, such as in finance and structural mechanics. Historically, mechanical constructions have been designed for predictable operation in the quasi-static regime and, more recently, in the harmonic regime. The ability to design structures for safe operation in the chaotic regime would push the envelope even further and pave the way for lighter and more flexible designs.

Much chaos research has dealt with small systems with attractor dimension close to phase space dimension. The Lorenz attractor [1] is perhaps the quintessential example: Originally a model of atmospheric dynamics, its phase space dimension is 3 and the attractor dimension is 2.06. However, in an effort to preserve quantitative accuracy, models in industrial use tend to contain very large numbers of variables. For example, “real” weather forecast models contain thousands to millions of variables. They are highly spatially resolved and the computational challenge of dealing with them is considerable. Yet, they still display low-D chaos [2]. Thus, here, the gap between phase space dimension (millions) and attractor dimension (single figures) is enormous.

While the seminal work of the likes of Poincaré and his colleagues laid a crucial foundation for the research in non-linear dynamics [3], Lorenz’s famous paper [1] was arguably the first complete account of a system exhibiting deterministic chaos. While the Lorenz equations, as a highly reduced version of the Navier-Stokes equations, described atmospheric dynamics, the range of systems now shown to display chaotic behaviour is vast. The decades that followed Lorenz’s paper saw the publication of a number of seminal chaos papers across several fields of science and the canon of chaos now includes systems derived from solid mechanics, fluid mechanics, electrical dynamics and chemistry [4–11]. Since the 1980s, in the wake of the expansion of mathematics into new fields, chaos research has come to interface with, e.g., finance, biology and medicine [12–23], but also with fields such as game theory, behavioural science and opinion formation [24–27].

In the early 1960s the Noble model [28, 29] of cardiac tissue was developed. The model was refined over the subsequent years and advanced incarnations of it are still being published today, with the most recent versions being relevant for drug discovery [30, 31] and as clinical decision tools [32]. What all Noble-type models have in common is that, being coupled systems of non-linear ODEs, they contain the substrate for chaos. Indeed, in the late 1980s it was shown experimentally that cardiac tissue, when driven at increasing pace, goes through a cascade of bifurcations and ultimately displays low-dimensional deterministic chaos [33]. While cardiology will probably be the first medical speciality to incorporate physiological and pathological mathematical models into the forefront of clinical practice, other areas of medicine are the subject of non-linear dynamics and chaos research, such as neurology [18, 19] and oncology [20, 34, 35].

Some of the more fundamental properties and definitions pertaining to chaos have begun to find use in practical applications. For example, the Lyapunov spectrum—a concept central to chaos—can be utilised for parameter identification purposes [36]. Also, the tendency of a chaotic system to visit large regions of state space can be exploited in control systems [37]. Luther and co-workers applied this principle to enable low energy resynchronisation of fibrillating cardiac myocytes [23] and managed to do so at significantly lower energies than those of standard defibrillation. Recently, Wang and co-workers developed a chaos control framework and applied it to control apoptosis in a model of T-cell leukaemia [21].

The search for methods to predict the future behaviour of chaotic systems spans at least three decades, but the only widely applicable methods thus far have focused on making tight and local near-future predictions [38, 39]. The advantage of such methods is that they delimit possible future states of a system relatively narrowly in phase-space and that they can be applied to high-D chaos in high-D phase space. The obvious drawback is their local and consequently short-term nature. The effort towards determining quantitatively accurate long-term global bounds of chaotic systems has mainly been focused on obtaining closed-form solutions to specific systems on an ad-hoc basis. As the information required to store a manifold is exponentially dependent on dimension, existing methods of bounding strange attractors break down in high-D phase space. Efforts to bound chaotic systems have, so far, mainly focused on systems of three variables [40, 41] with attractor dimension close to phase space dimension. A more generalised method for the bounding of low-D chaotic systems was proposed in [42, 43], which took the approach of defining localising sets with bounding manifolds defined as polynomials.

The present method is applicable in situations when the phase-space is high-D, but the attractor is low-D. In this situation it is computationally feasible to define a long-term bound for the system. Although study of high-D chaos and the factors impacting on attractor dimension has taken place since the 1980s [39, 44–46], as discussed above, many natural processes, even if high-D in terms of phase space, generate low-D attractors. It has been and still is commonplace to reduce large systems to the minimum dimension necessary for *qualitative* accuracy [5], which in turn tends to be defined as preservation of the topology of the attractor [9]. However, qualitative methods sacrifice quantitative accuracy. And quantitative accuracy may be crucial. Even periodic systems, are often simulated by very large quantitatively accurate high-D models. The study of dynamic phenomena with linear finite element models is an example of this.

The method presented here finds its niche in situations where model reduction is unacceptable and the extrema of system variables are important. It is deliberately designed to be intuitive and easy to apply, i.e. “wrap around” an existing system. The aim is that, once applied and proven on attractors of higher (but still low) dimension, it can be used by non-experts in chaos and non-linear dynamics in a plug and play fashion.

## Results

Two manifolds are defined in the following: The auxiliary manifold (AM) and the bounding manifold (BM). The AM is an *M*-dimensional manifold, where *M* is an integer. It is embedded in the phase space of the system to be bounded and, in turn, the BM is embedded on the AM. The hope is that the AM and the BM display stationary harmonic behaviour in time, such that they only need to be calculated for a couple of post-transient forcing cycles.

### The bounding- and auxiliary manifolds

The manifolds are non-autonomous extensions of the concept of invariant and inertial manifolds and draw inspiration from existing literature [47, 48]. However, the following does not adhere strictly to the quite specific definitions and nomenclature of inertial manifold theory. Firstly, inertial manifolds are most often employed as a means to directly solve the original equations *on the manifold*. They allow the reduction of dimensionality from either infinity (for partial differential equations) or high-finite dimension (ODEs with many degrees of freedom). Here, no attempt is made at solving the underlying system of ODEs on the manifolds. The AM serves the purpose of reducing the dimensionality of the BM—not that of the underlying problem. Secondly, inertial manifold theory rests on the premise that solutions in full phase-space approach *a solution on* the manifold. The requirement here is simply that solutions approach the manifold, which is less restrictive. So, although the AM draws from inertial manifold theory, it is named as it is to emphasize the differences and avoid confusion.

If all unstable directions are contained on the AM, solutions that do not start out on the manifold, will decay exponentially onto it and, post-transiently, an attractor will lie on the AM. Let **c** denote the bounding manifold. Then, the premise of the method is that

$$\begin{array}{cc} & A\subset C\subset B\\ & c=\partial C \end{array}$$ (1)

where *A* is an attractor, *B* is the auxiliary manifold and *C* is an *M*-manifold with boundary. Thus, **c** is a closed (*M* − 1)-manifold. Note that “bounding manifold” is formally a (potential) misnomer as extrema of the system may lie on interior points (and not on the BM itself). Further, this necessitates the post hoc calculation of interior points, which is discussed below.

The AM is embedded in the full phase space $x\in R^{N}$ of the system of ODEs:

$$\begin{array}{c} \dot{x}=f\left(x,t\right) \end{array}$$ (2)

**Fig 1:**
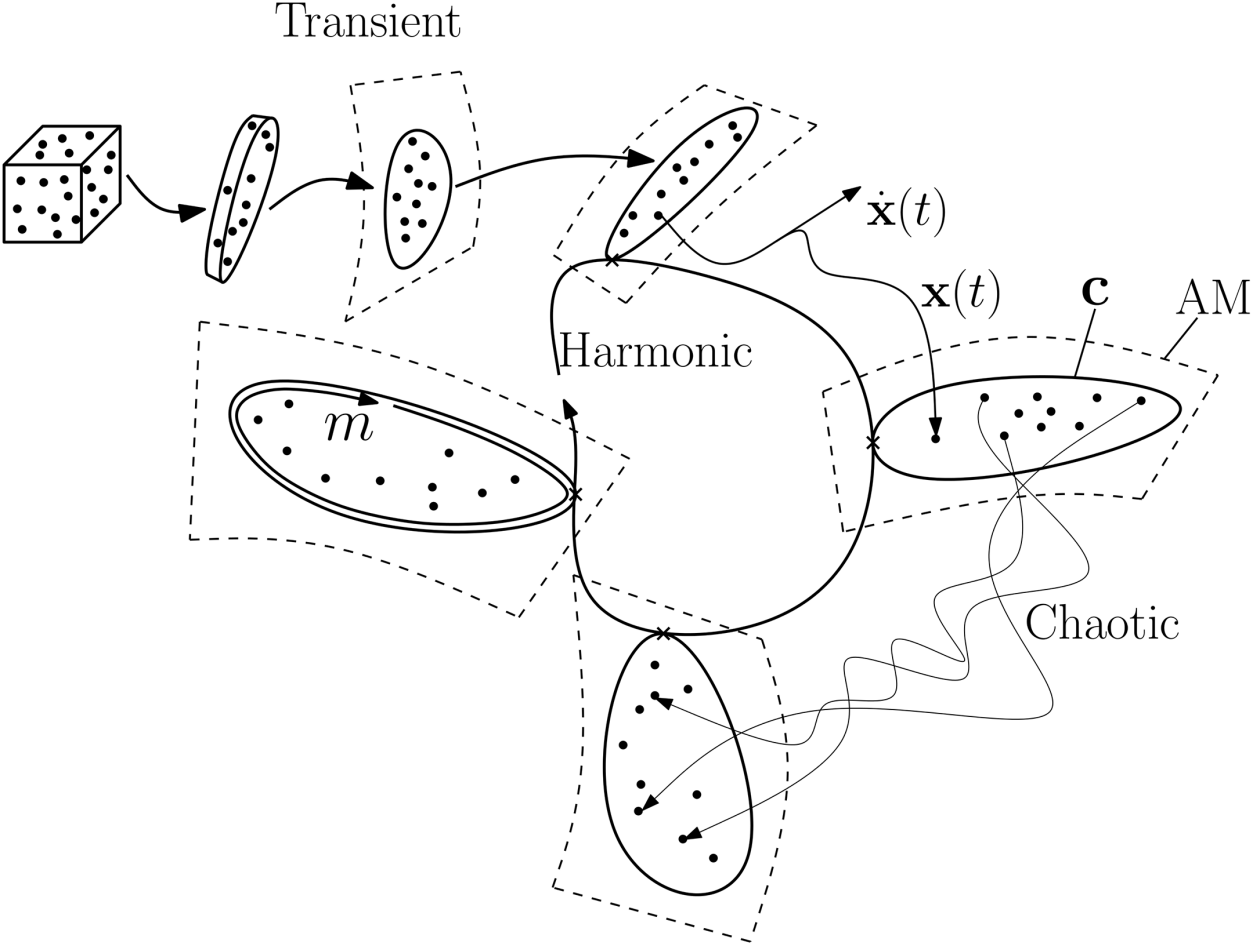
Schematic showing the contraction of a set of initial conditions along off-manifold directions onto the AM and the post-transient harmonic behaviour of the AM and BM. The parameter *m* runs from 0 to 1 along the length of the BM. A number of solutions are shown as dots. Initially they lie as a cloud in phase space, but they rapidly contract along stable directions until they converge to the AM. As shall become apparent, it is sufficient to calculate the tangent space of the AM to orientate the BM **c**(*m*, *t*). This is done with a spectral method.

As it shall become apparent in the following, it is a requirement that **f** is smooth. Furthermore, it is a requirement that *M* > *d*, where *d* is the (fractal) dimension of the attractor. The most obvious choice is to select *M* as the nearest integer greater than *d*. As mentioned above, the discipline of bounding chaotic systems has previously focused on *N* − 1 dimensional boundaries to systems with *N* dimensional phase spaces [40, 42, 43]. Such approaches will fail rapidly as *N* increases to that of even modest sized problems, since the computational cost of operating on a discretised manifold depends exponentially on its dimension. Here, it is exploited that the strange attractor is normally a low-D entity in a high-D phase space, i.e, that *d* < *M* < <*N*. So, the BM, **c**(*t*), need not be more than *M* − 1 dimensional. If **c**(*t*) is harmonic in time, the solution can be stopped after a couple of post-transient forcing cycles. Fig 1 shows this scenario for *M* = 2. For the method to work, the AM must be invariant under the flow, meaning that solutions that start on it, stay there. The manifold is allowed to vary with time. So, labelling the manifold “invariant” may cause confusion. Nevertheless, this is the naming convention widely adopted in literature and time dependent inertial manifolds are well known [49]. The key requirement is that the manifold displays regular motion. Clearly, if the manifold itself is chaotic, hence the BM also, the method has achieved very little.

**Fig 2:**
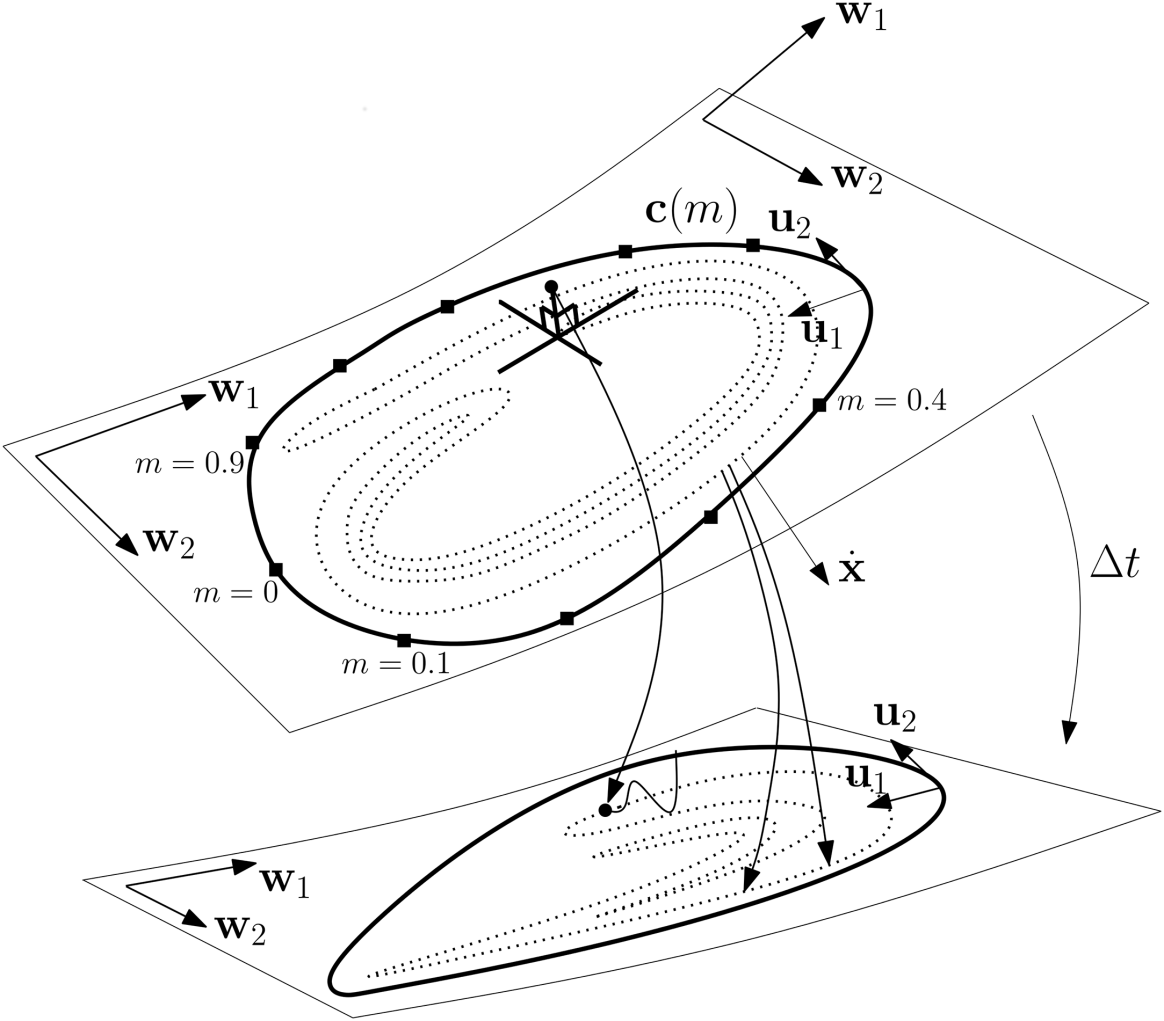
Schematic of AM and BM. An off-manifold point with stable orthogonal motion is shown. The unit vector **u**_1_ points in the inward direction and the unit vector **u**_2_ is tangential to the BM. The vectors **w**_1_(**x**) and **w**_2_(**x**) span the tangent space of the AM. On the BM (**u**_1_,**u**_2_) and (**w**_1_,**w**_2_) span the same 2-D tangent space. Here, the dimension of the AM, *M*, is 2.

To introduce key concepts, we consider the case of a 2-D AM in a $R^{N}$ phase space and then generalise the ideas to *M*-D manifolds in $R^{N}$ phase spaces. Fig 2 shows a schematic of the BM and AM. It also introduces the basis vectors of the AM (**w**_1_,**w**_2_), the inward normal **u**_1_ and the BM unit tangent vector **u**_2_. Furthermore, the figure shows an off-manifold point which is projected along a stable direction onto the manifold. If certain criteria are met, it can be proven that the off-manifold point converges exponentially with a solution on the manifold [9, 48, 50, 51]. However, what matters is that off-manifold points approach the manifold asymptotically, end up *somewhere* on it and stay there, which is uncontroversial. Here, the notion of points being “on” the manifold is taken to include points infinitesimally close to it, which is the case with asymptotically approaching points. Obviously, once the distance drops below machine precision, there is no practical distinction.

The motion of the BM is the sum of the tangent on-manifold motion and the orthogonal off-manifold motion. The latter is readily determined through Gram-Schmidt processing of the flow. The expression for BM motion then becomes:

$$\begin{array}{c} \dot{c}=\underset{⊥}{\underset{︸}{f-u_{1}\left(f\cdot u_{1}\right)-u_{2}\left(f\cdot u_{2}\right)}}+\underset{∥}{\underset{︸}{u_{1}d_{1}+u_{2}d_{2}}} \end{array}$$ (3)

where **f**(**x**, *t*) = **f**(**c**(*m*), *t*) is defined in Eq (2), $\dot{c}\left(m,t\right)$ is the rate of the BM, *d*_*i*_ are the on-manifold velocities and **u**_*i*_ is a local orthonormal basis spanning the tangent space of the AM. For AMs of dimension 3, 4 and so on, Eq (3) is easily expanded with *d*_3_, **u**_3_, *d*_4_, **u**_4_, etc. That is, generally

$$\begin{array}{c} \dot{c}=\underset{⊥}{\underset{︸}{f-\sum_{i=1}^{M}u_{i}\left(f\cdot u_{i}\right)}}+\underset{∥}{\underset{︸}{\sum_{i=1}^{M}u_{i}d_{i}}} \end{array}$$ (4)

The flow and the vectors are evaluated locally along the length of the BM and depend on time, i.e.

$$\begin{array}{cc} & f=f(x,t)=f(c(m),t)\\ & u_{i}=u_{i}\left(c\left(m\right),t\right),\;i=1,2,...,M\\ & d_{i}=d_{i}\left(c\left(m\right),t\right),\;i=1,2,...,M \end{array}$$ (5)

The fundamental idea behind the method is that the locally unstable directions, the directions of stretch, are contained in the on-manifold terms (“∥”). If that is the case, the remaining off-manifold motion (“⊥”) will consist only of exponential decay onto the manifold.

The nomenclature is such that **u**_1_ is the inward normal (tangent to the AM and orthogonal to the BM) with all other vectors spanning the space tangent to the BM (tangent to both the AM and the BM).

The inward motion of the boundary is taken to follow the heuristic expression:

$$\begin{array}{c} d_{1}=\max\left(\;u_{1}\cdot f,\;\kappa /\beta \right) \end{array}$$ (6)

where *β* is the linear impedance and *κ* = *κ*(**c**(*m*, *t*)) is the signed dimensionless curvature signal passed through a low-pass filter with time constant *τ*:

$$\begin{array}{c} \dot{\kappa }=\left(\frac{\partial u_{2}}{\partial m}\cdot u_{1}-\kappa \right)/\tau \end{array}$$ (7)

The equation of motion Eq (6) automatically bounds the underlying ODE and filters away high frequency oscillations. It will straighten the BM locally until the curvature term vanishes, at which point the BM is at equilibrium. Since the BM is a closed curve, the global net effect of the curvature term is to contract the BM. The responsiveness of the BM is determined by the values of *β* and *τ*. Although the expression is non-linear in *d*_1_, it has the merit of being local, so does not require the (iterative) solution of a system of non-linear equations. A spatially coupled formulation, e.g., based on constrained optimization and a Lagrange multiplier field, would come with this drawback. The present study considers 1-D BMs, so the measure of curvature is given. For higher-dimensional BMs (2-D surfaces and beyond), a scalar quantity such as the mean curvature might be used. The motion of the BM is discretised using Hermite finite elements. These have the sufficient order to allow the calculation of curvature.

#### Axial motion

The axial motion of the BM seeks to space nodes evenly along the BM. This is no more than an adaptive meshing exercise. For the sufficiently well discretised BM, the axial motion has no appreciable impact on the shape of **c**. The below expressions are valid for a 1-D BM, so a general (*M* − 1)-D version is needed. Consider the rate of change of elastic energy with the addition of a dissipative term

$$\begin{array}{c} s=\left(c^{\prime }\cdot u_{2}\right)\left({\dot{c}}^{\prime }\cdot u_{2}\right)+\frac{1}{2}\beta_{2}d_{2}^{2} \end{array}$$ (8)

Now, applying Eq (3) and requiring stationarity with respect to the *d*_2_ field, one gets the expression

$$\begin{array}{c} \delta s=\left(c^{\prime }\cdot u_{2}\right)u_{2}\cdot \left(u_{2}\delta d_{2}^{\prime }+u_{2}^{\prime }\delta d_{2}\right)+\beta_{2}d_{2}\delta d_{2}=0 \end{array}$$ (9)

where prime denotes differentation with respect to *m*. As with the *d*_1_ field described above, the *d*_2_ field is expanded in terms of Hermite shape functions and the finite element method is used to solve Eq (9) for nodal values of *d*_2_. The details are given in the methods section.

### Interior points

Unlike in some other methods from the literature, the interior of the manifold is not necessary for the determination of manifold orientation. Despite this, it must still be considered, as an extremum of a state variable might lie on the interior and not on the BM. One must balance the added computational cost of sampling the interior against the need for a high number of samples. The equation of motion for the interior points is a compromise between simplicity and the desire for evenly spaced points and could be defined in a variety of ways. What is unequivocal though, is that interior points must ride on the tangent space associated with the AM.

The orientation at interior points is determined in the same way as on the BM. A practical and fairly straight forward way of ensuring evenly spaced points throughout a simulation, where the BM may change shape significantly, is to connect the interior points to their neighbours and the BM with linear truss elements of zero initial length. Then, point positions are updated dynamically as nodes of truss elements. For the purposes outlined here, this is nothing more than a simple, and probably sub-optimal, adaptive meshing technique. The advantage of truss elements is that they are readily defined in higher dimensions as their length can be easily calculated in N-dimensional Cartesian coordinates. Thus, they are practical for point spacing in phase spaces above 3-D. The equation of motion for interior points is identical in form to Eq (3), except that now *d*_1_ and *d*_2_ are determined by force summations of the truss elements. That is, the interior points, i.e., truss element nodes, are restricted to move on the AM onto which all truss element forces, accelerations and velocities are projected. The truss element normalised stiffness and damping, *k*_*t*_/*m*_*t*_ and *d*_*t*_/*m*_*t*_, respectively, are given in Table 1. The motion of truss element nodes on the BM **c** is prescribed to follow the BM in a one-way coupling fashion, so the motion of the BM is not influenced by the interior nodes. This means that the motion of the BM can be computed first, and interior point sampling performed in post-processing.

**Table 1:** Model parameters for the simulations. The parameter *τ* is the time constant for curvature filtering, *β* is the proportional impedance on curvature, *β*_2_ is the impedance on axial motion, *k*_*t*_, *d*_*t*_ and *m*_*t*_ are the stiffness, damping and mass of interior point truss elements, *α* is the non-linear Duffing stiffness, *k*_1_, *d*_1_ and *m*_1_ are the linear stiffness, damping and mass of the Duffing oscillator, *f*_0_ is the forcing term amplitude, *ω* is the forcing term frequency, *k*_2_, *d*_2_ and *m*_2_ are the stiffness, damping and mass of the linear oscillators, Δ*t* is the time step for the time integration scheme (BM and interior point position update), Δ*t*_*λ*_ is the time step for eigenvalue calculation and orientation update, *K* is the number of nodes (and elements) used to discretise **c**, and *K*_*G*_ is the number of Gauss points for numerical integration in space along **c**.

All	*τ* = 0.01	*β* = 0.1	*β*_2_ = 1.0	*k*_*t*_/*m*_*t*_ = 500
	*d*_*t*_/*m*_*t*_ = 50	*α* = 1	*k*_1_ = −1	*m*_1_ = 1
	Δ*t* = *π*/2 ⋅ 10^−5^	Δ*t*_*λ*_ = *π* ⋅ 10^−2^	*K* = 80	*K*_*G*_ = 280
I	N = 22	*f*_0_ = 1.0	*ω* = 2.0	*d*_1_ = 0.2
	*m*_2_ = 0.05	*d*_2_ = 2.0	*k*_2_ = 20.0	
II	N = 42	*f*_0_ = 0.5	*ω* = 1.0	*d*_1_ = 0.2
	*m*_2_ = 0.025	*d*_2_ = 2.0	*k*_2_ = 20.0	
III	N = 82	*f*_0_ = 0.5	*ω* = 1.0	*d*_1_ = 0.2
	*m*_2_ = 0.0125	*d*_2_ = 4.0	*k*_2_ = 40.0	
IV	N = 22	*f*_0_ = 0.4	*ω* = 1.0	*d*_1_ = 0.1
	*m*_2_ = 0.05	*d*_2_ = 2.0	*k*_2_ = 20.0	

The equation of motion for an interior point, $o\in R^{N}$, is

$$\begin{array}{c} \dot{o}=\underset{⊥}{\underset{︸}{f-\sum_{i=1}^{M}w_{i}\left(f\cdot w_{i}\right)}}+\underset{∥}{\underset{︸}{\sum_{i=1}^{M}w_{i}d_{i}}} \end{array}$$ (10)

where *d*_*i*_ are determined as

$$\begin{array}{c} a_{i}=\sum_{i}w_{i}\cdot h,\;d_{i}=\int_{t}a_{i}dt \end{array}$$ (11)

and $h\in R^{N}$ is the resultant mass-normalised force from trusses pulling on an interior point.

### Example: The duffing oscillator

The Duffing oscillator is among the simplest systems known to exhibit chaos, yet still finds use for a wide variety of applications including, e.g., nano-mechanical systems [52]. Consider a system consisting of a Duffing oscillator in series with a number of linear spring-mass-damper oscillators. The linear oscillators add to the phase space of the system, and quantitatively affect the solution to the equations, but do not add to the dimension of the attractor or influence its topology. If the duffing oscillator is assigned the first two degrees of freedom, the system of equations can be written as

$$\begin{array}{cc} & {\dot{y}}_{1}=y_{2}\\ & {\dot{y}}_{2}=\frac{1}{m_{1}}\left(f_{0}\cos\left(\omega t\right)-k_{1}y_{1}-d_{1}y_{2}-\alpha y_{1}^{3}+\left(y_{3}-y_{1}\right)k_{2}+\left(y_{4}-y_{2}\right)d_{2}\right)\\ & {\dot{y}}_{3}=y_{4}\\ & {\dot{y}}_{4}=\frac{1}{m_{2}}\left(\left(y_{1}-2y_{3}+y_{5}\right)k_{2}+\left(y_{2}-2y_{4}+y_{6}\right)d_{2}\right)\\ & ...\\ & {\dot{y}}_{2i-1}=y_{2i}\\ & {\dot{y}}_{2i}=\frac{1}{m_{2}}\left(\left(y_{i2-3}-2y_{2i-1}+y_{2i+1}\right)k_{2}+\left(y_{i2-2}-2y_{2i}+y_{2i+2}\right)d_{2}\right),i=3,4,..\\ & ...\\ & {\dot{y}}_{N-1}=y_{N}\\ & {\dot{y}}_{N}=\frac{1}{m_{2}}\left(\left(y_{N-3}-y_{N-1}\right)k_{2}+\left(y_{N-2}-y_{N}\right)d_{2}\right) \end{array}$$ (12)

where *f*_0_cos(*ωt*) is a harmonic forcing term, *k*_1_, *d*_1_, *α* and *m*_1_ are the parameters of the Duffing oscillator and all the linear oscillators are assigned the identical parameters *k*_2_, *d*_2_ and *m*_2_. The governing equations are on the form of Eq (2) so can be plugged into the method as described above. In order to reduce the mesh refinement and time step requirements associated with curvature and stretch along fast, linear terms, the equations are transformed as **x** = [*x*_1_, *x*_2_, *x*_3_, …, *x*_*N*_]^*T*^ = [*y*_1_, *y*_2_, *y*_3_/10, …, *y*_*N*_/10]^*T*^ and transformed back in post-processing. This transformation is beneficial and possible because it is known a-priory that the non-linearity of the system appears in the first two equations—thus “compressing” all other variables and effectively increasing mesh refinement in the non-linear directions. For some systems, all variables might appear in non-linear terms, or it may not be known which ones that do. Then, scaling becomes futile, potentially necessitating finer meshes and shorter time steps. Parameters for all simulations are shown in Table 1. In general terms, a fast system requires a fast BM. So a system containing high frequencies and rapid changes in **J** will require low impedances on the BM, lower time constants for the first order filter and, of course, smaller time steps.

**Fig 3:**
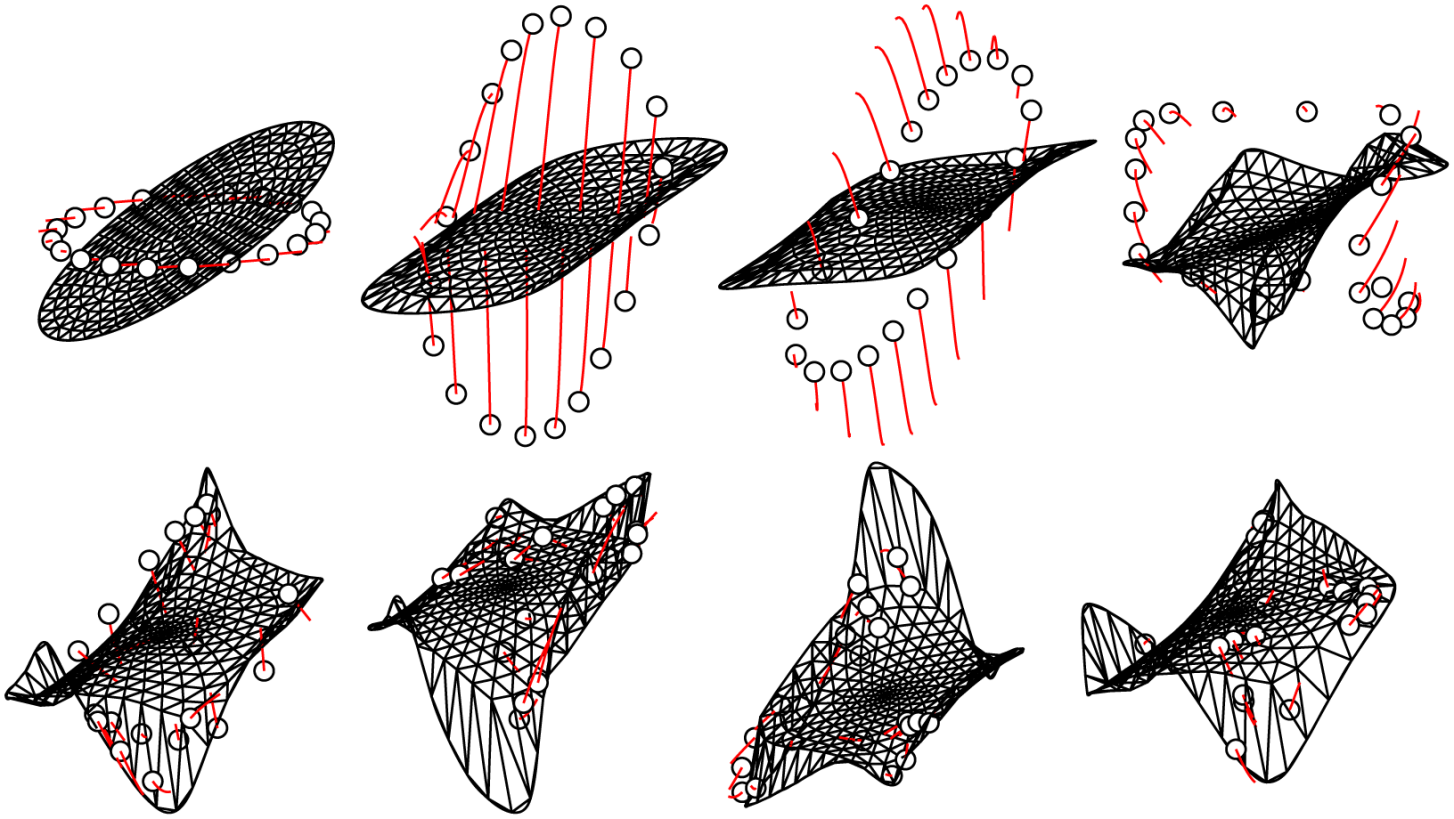
The (*y*_1_, *y*_2_, *y*_42_) projection of the AM and BM at times *tω*/(2*π*) = {0.005, 0.1, 0.15, 0.3, 0.6, 1.5, 2.0, 4.66} during a simulation. Overlaid are twenty different time series with tails tracing their motion shown in red. Each tail represents Δ*tω*/(2*π*) = 0.05. The time series rapidly converge onto the manifold. The BM will expand to contain those attractors whose basins of attraction it intersects. The interior points are connected with truss elements of zero initial length which are shown in the figure.

**Fig 4:**
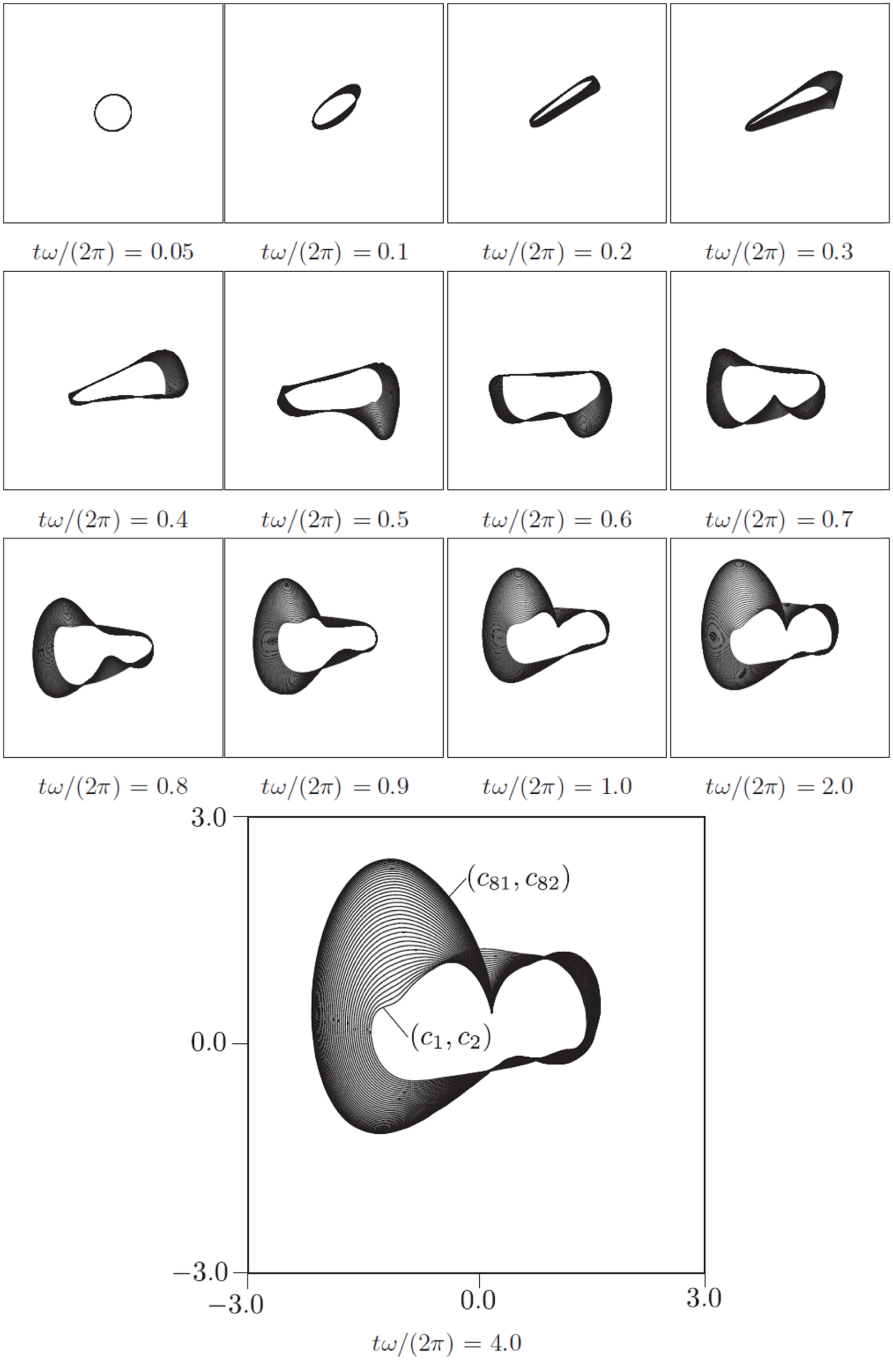
Snapshots of projections of the BM c(*t*) onto coordinate surfaces (*y*_1_, *y*_2_), (*y*_3_, *y*_4_), …, (*y*_81_, *y*_82_) for various times during a simulation for parameter set III in Table 1. Note that the last two figures are largely similar, indicating that the BM is becoming post-transient.

In Fig 3 is shown the convergence of the AM with twenty solutions for parameter set II in Table 1. The phase space is 42-*D*, and only the (*y*_1_, *y*_2_, *y*_42_) projection is shown, so more information is contained in the simulation than can be displayed on the figure. Note that the solutions converge to the AM and stay there. The figure also shows the truss elements connecting interior points to each other and to the BM. A video of a similar process for parameter set III is shown in S1 File. Fig 4 shows snapshots of projections of the BM onto a selection of coordinate surfaces for parameter set III in Table 1. The projections vary considerably in shape and size, indicating differences in phase and magnitude, respectively, of the harmonic components of the state variable motion. A video of a similar process for parameter set III is shown in S2 File.

**Fig 5:**
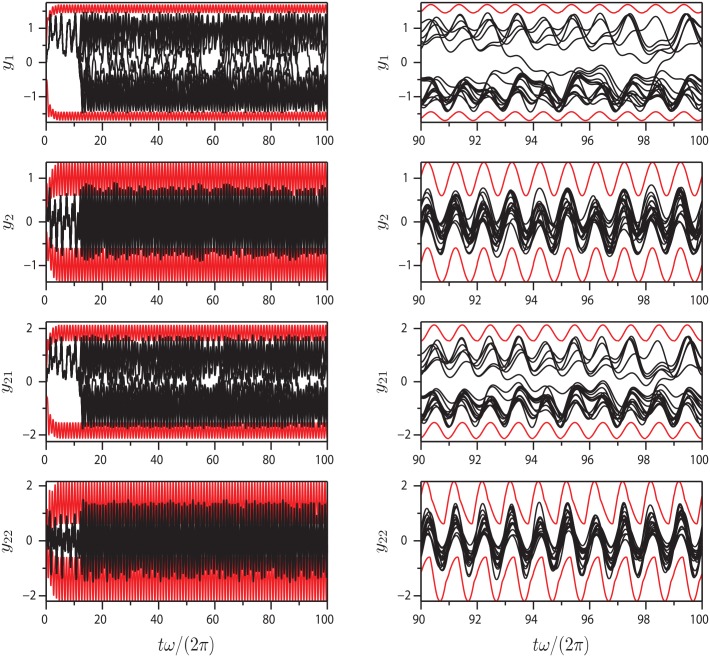
Extrema of Eq (12): Plots of extrema (red) and 20 different time series (black) of variables *y*_1_, *y*_2_, *y*_21_ and *y*_22_ for parameter set I. The bounds for odd (0th order) variables are somewhat tighter than those of even numbered (1st order) variables.

**Fig 6:**
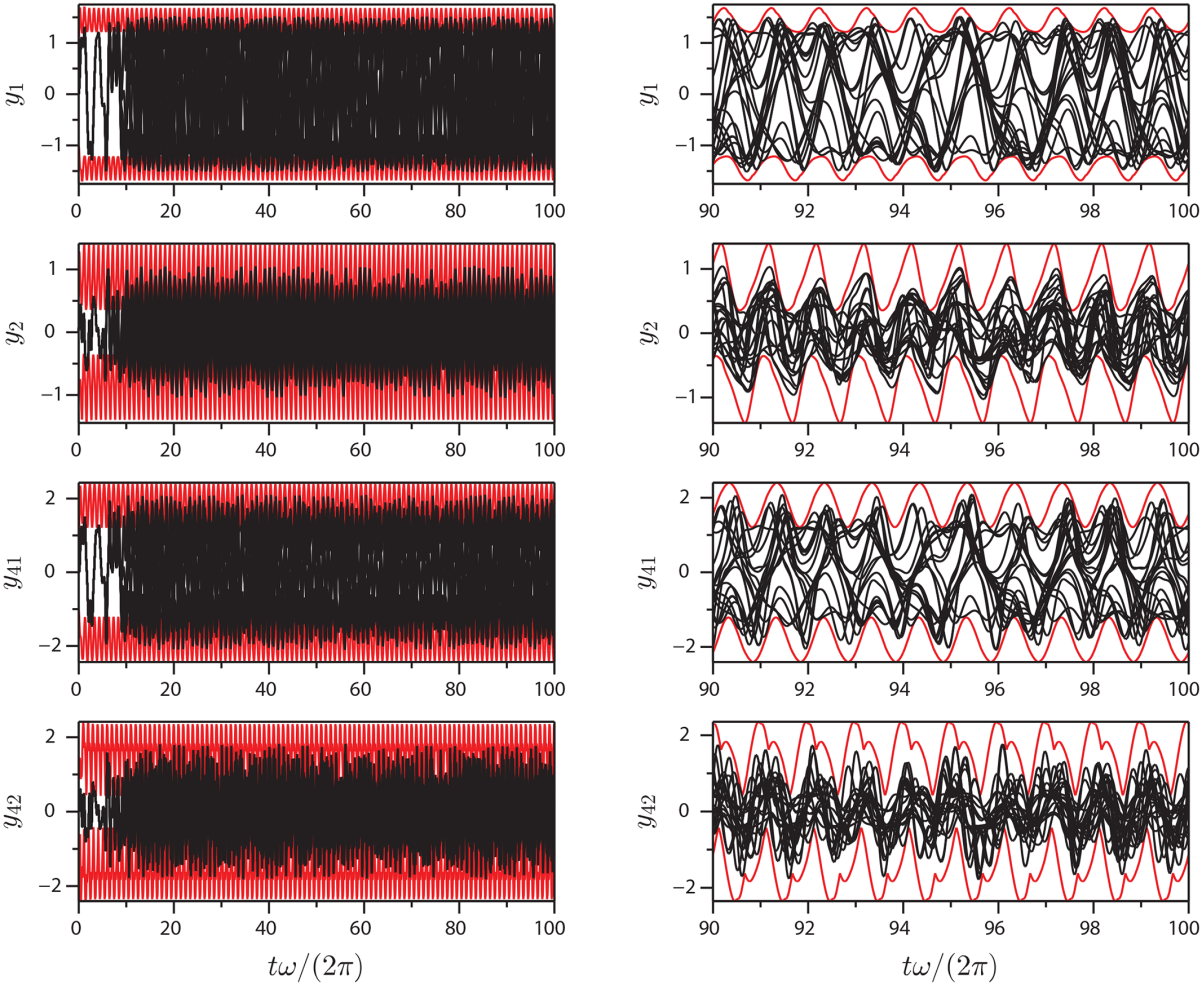
Extrema of Eq (12): Plots of extrema (red) and 20 different time series (black) of variables *y*_1_, *y*_2_, *y*_41_ and *y*_42_ for parameter set II.

**Fig 7:**
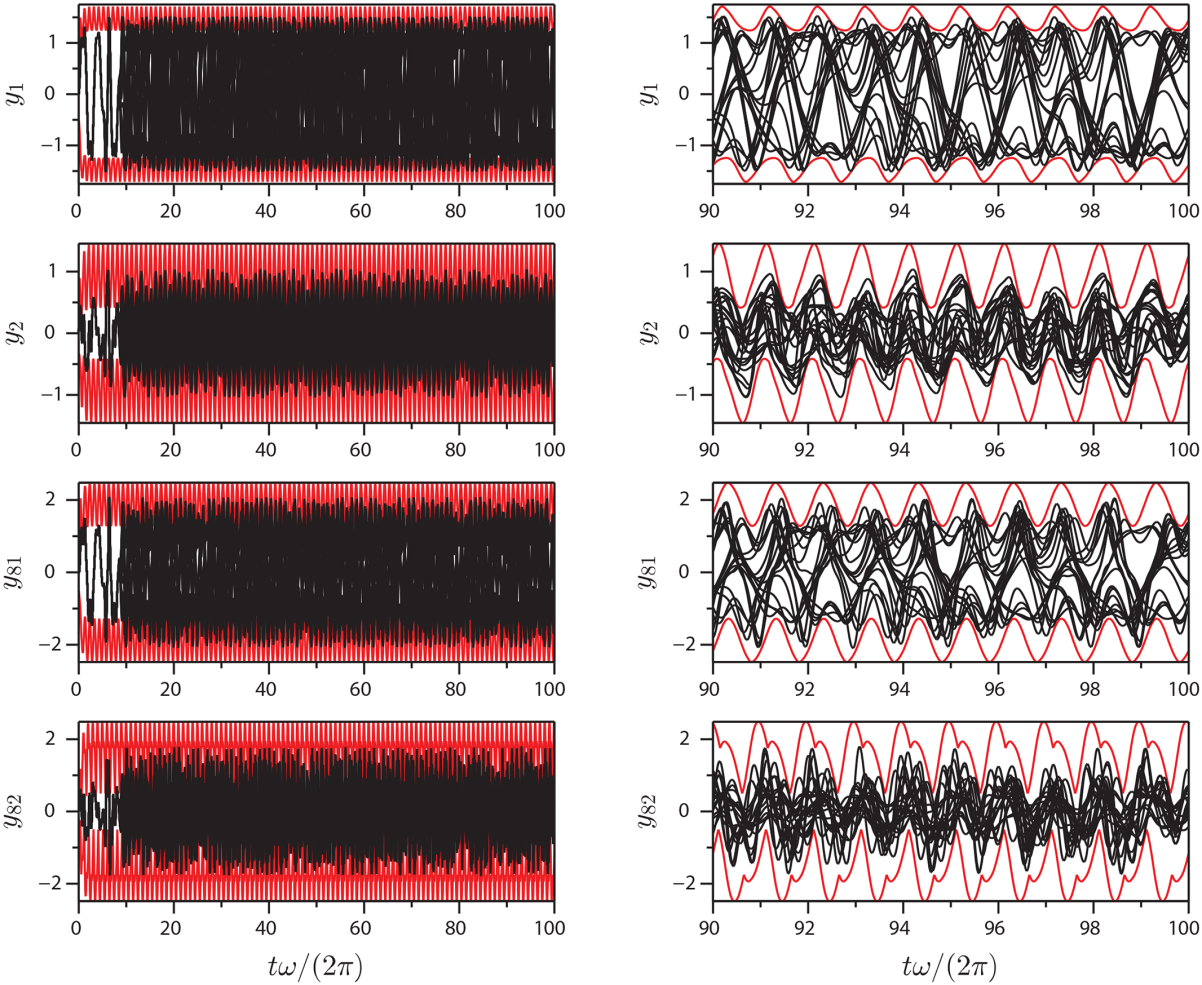
Extrema of Eq (12): Plots of extrema (red) and 20 different time series (black) of variables *y*_1_, *y*_2_, *y*_81_ and *y*_82_ for parameter set III.

Figs 5–7 show projections of extremal values onto selected coordinate directions (*y*_1_, *y*_2_, *y*_*N*−1_ and *y*_*N*_) for parameter sets I-III. These are found by sampling all Gauss points on the BM as well as the interior points (see, e.g., Fig 3). The motion of the AM and the BM is calculated for five forcing cycles and the subsequent 95 cycles are copies of the fifth, which is post-transient. Twenty time series are calculated for 100 forcing cycles in order to validate the BM and it is seen that they all remain within the predicted values. The BM is noted to be reasonably tight and to be following the low frequency dynamics of the system. As one would expect, not all variables in the system influence the BM equally. The geometric nature of the method means that for the BM to contract, curvature must exist in the coordinate direction of the relevant variable. With the proposed equation of motion, the BM will tend towards being convex everywhere. The approach works well with the here presented Duffing-type system, but there is no proof that it will in general, so future modifications to the equation of motion Eq (6) producing a locally concave BM might be beneficial.

### Example: The parametric duffing oscillator

To illustrate the method’s ability to deal with situations where the Jacobi matrix depends explicitly on time, a parametrically excited version of the duffing equation is considered. The equations for this system are given in Eq (13). Notice that the non-linear term now varies explicitly with time.

$$\begin{array}{cc} & {\dot{y}}_{1}=y_{2}\\ & {\dot{y}}_{2}=\frac{1}{m_{1}}\left(-k_{1}y_{1}-d_{1}y_{2}-\left(\left(1-f_{0}\right)+f_{0}\cos\left(\omega t\right)\right)\alpha y_{1}^{3}+\left(y_{3}-y_{1}\right)k_{2}+\left(y_{4}-y_{2}\right)d_{2}\right)\\ & {\dot{y}}_{3}=y_{4}\\ & {\dot{y}}_{4}=\frac{1}{m_{2}}\left(\left(y_{1}-2y_{3}+y_{5}\right)k_{2}+\left(y_{2}-2y_{4}+y_{6}\right)d_{2}\right)\\ & ...\\ & {\dot{y}}_{2i-1}=y_{2i}\\ & {\dot{y}}_{2i}=\frac{1}{m_{2}}\left(\left(y_{i2-3}-2y_{2i-1}+y_{2i+1}\right)k_{2}+\left(y_{i2-2}-2y_{2i}+y_{2i+2}\right)d_{2}\right),i=3,4,..\\ & ...\\ & {\dot{y}}_{N-1}=y_{N}\\ & {\dot{y}}_{N}=\frac{1}{m_{2}}\left(\left(y_{N-3}-y_{N-1}\right)k_{2}+\left(y_{N-2}-y_{N}\right)d_{2}\right) \end{array}$$ (13)

**Fig 8:**
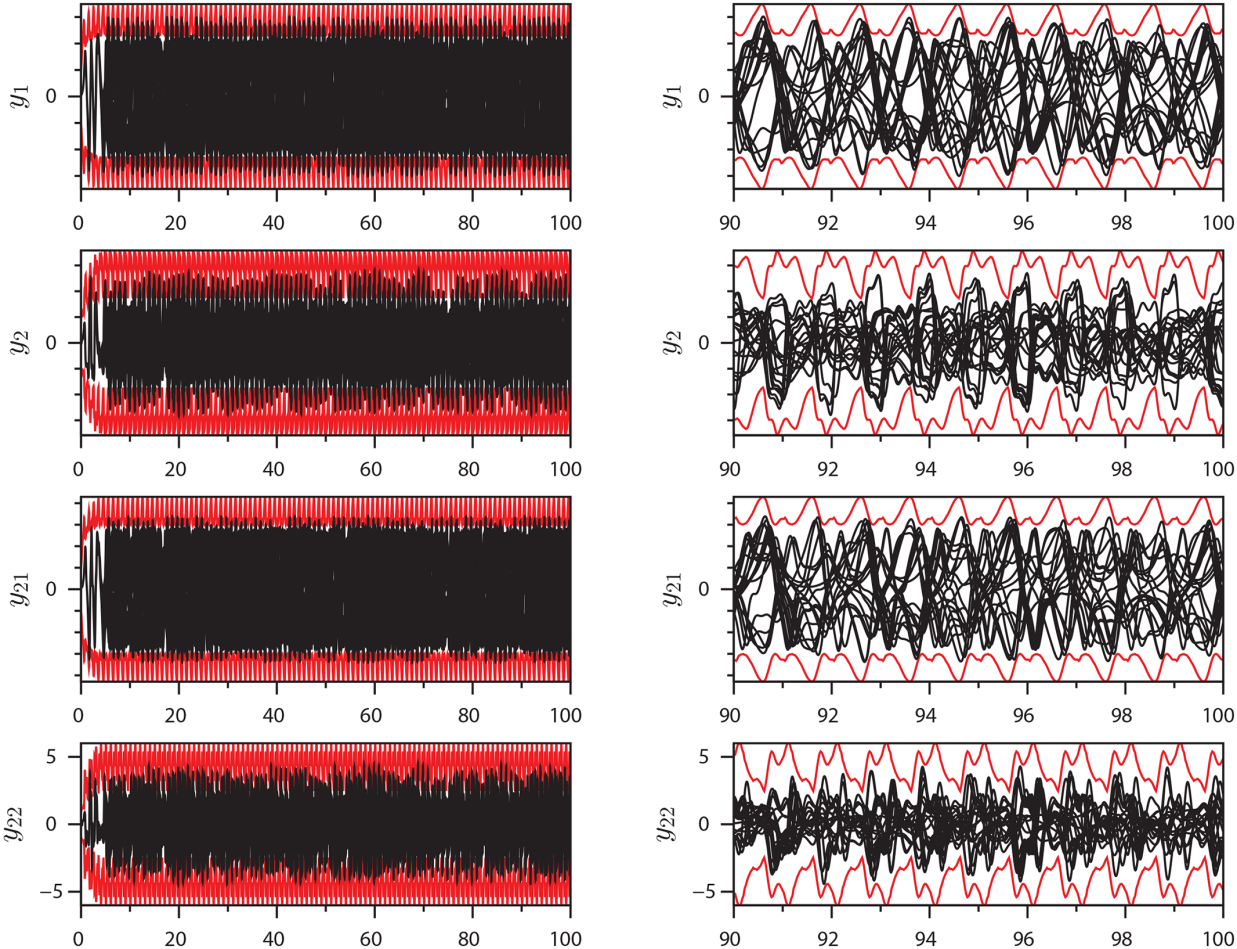
Extrema of Eq (13): Plots of extrema (red) and 20 different time series (black) of selected variables for parameter set IV.

**Fig 9:**
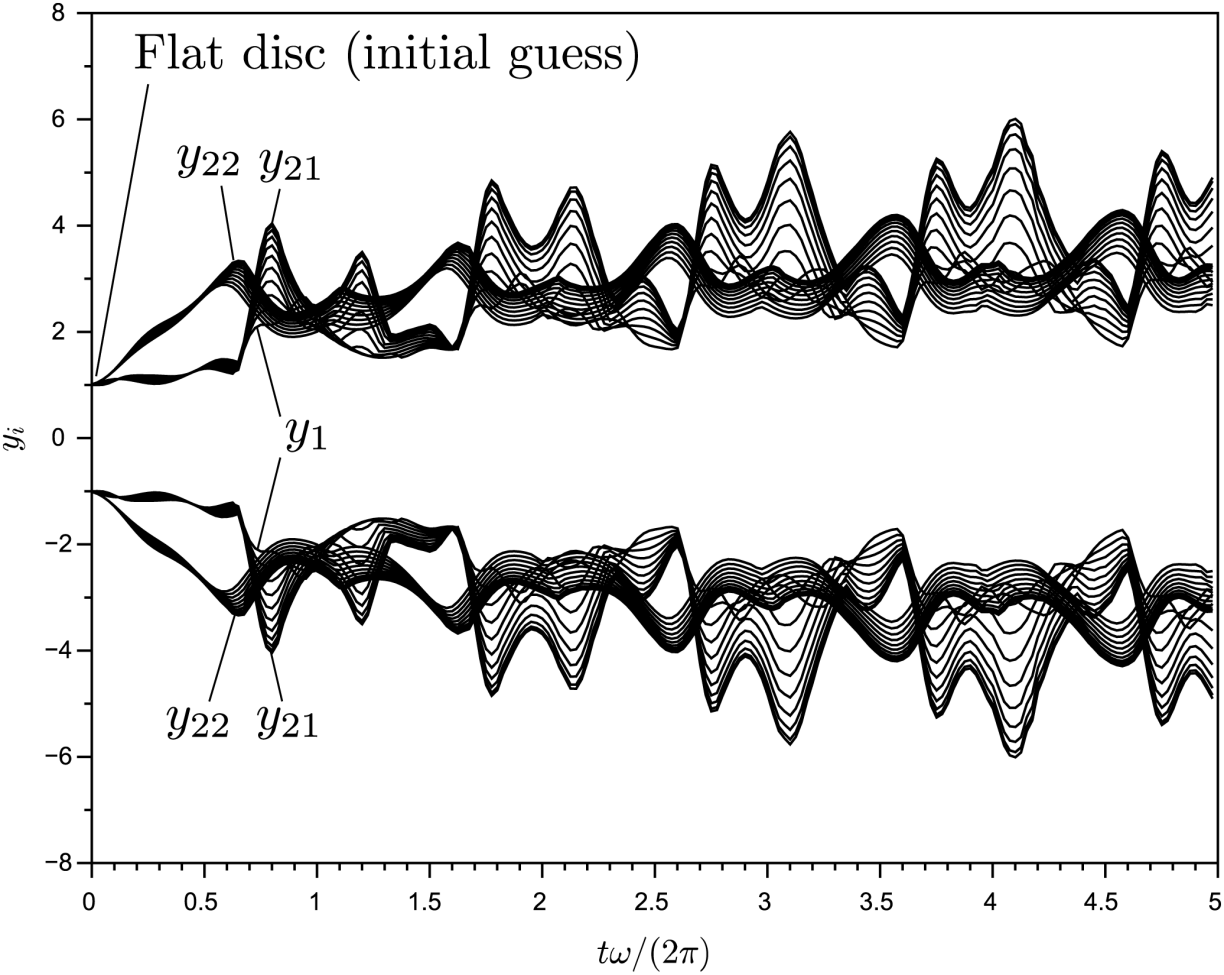
Transient phase of extrema calculated for Eq (13) (parameter set IV). The extrema will not be valid before the manifold is post-transient.

This system has an explicitly time dependent Jacobi matrix. Fig 8 shows the extrema of Eq (13) with parameter set IV as computed by the method. The method bounds the equation successfully. This is as expected, since the underlying derivations make no assumptions of autonomy: See the methods section for details. Fig 9 shows the transient phase of the extrema with indication of some of the degrees of freedom.

## Methods

### Local and instantaneous stretch and compression

**Fig 10:**
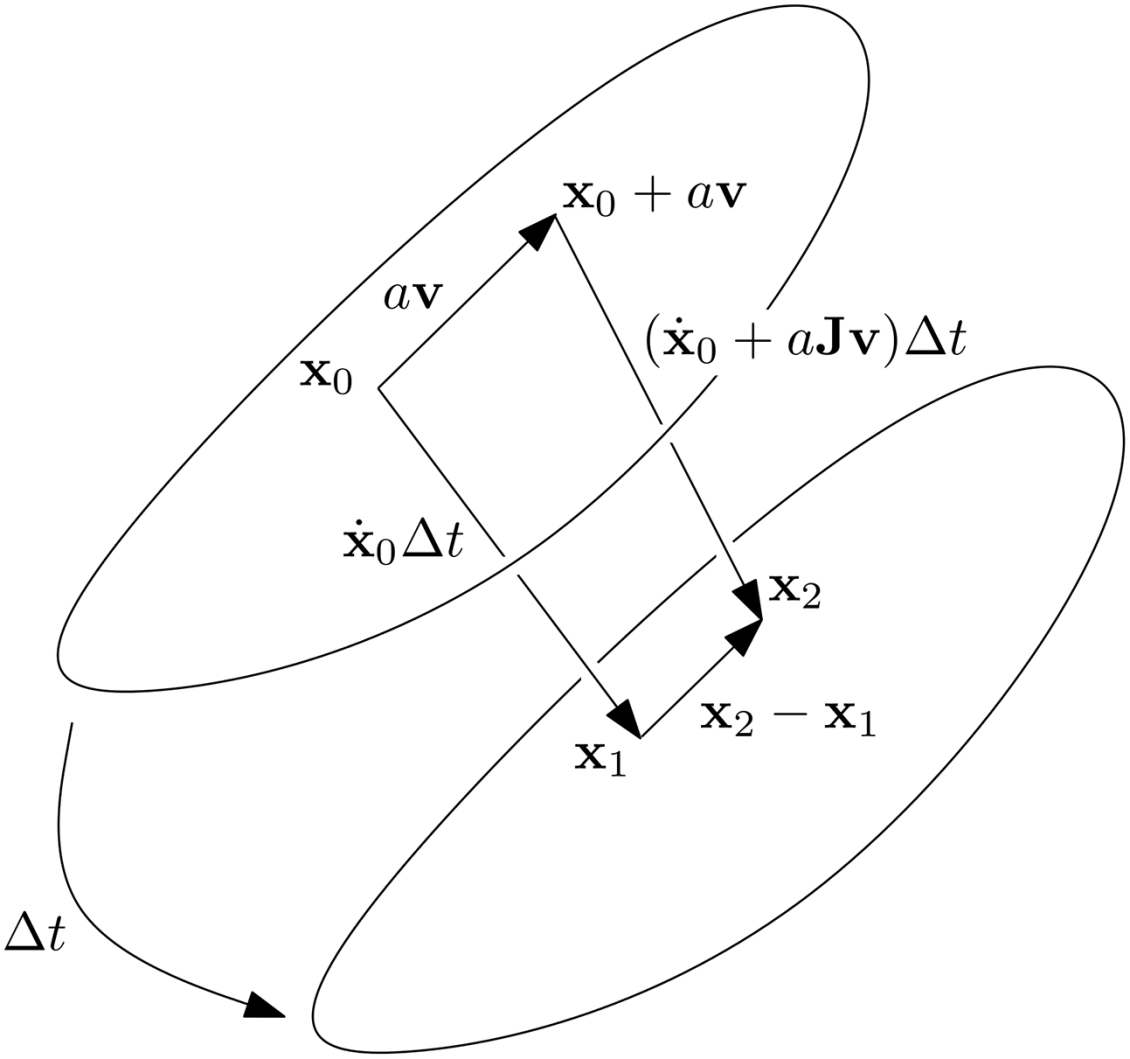
Perturbed solutions and their behaviour. These particular solutions are shown to converge as time is perturbed forward. The *N*–dimensional state space is represented conceptually as a surface and time as an arrow.

Let us explore the behaviour of two infinitesimally nearby trajectories in a smooth, non-linear and non-autonomous flow. Due to smoothness, the state can be represented by its Taylor expansion. Consider a perturbation in time by Δ*t* and in space by *a* **v**, where a is *a* scalar and $v\in R^{N}$ is a unit vector:

$$\begin{array}{c} x=x_{0}+\dot{x}\Delta t+\frac{\partial x}{\partial a}a+\frac{1}{2}\ddot{x}\Delta t^{2}+\frac{1}{2}\frac{\partial^{2}x}{\partial a^{2}}a^{2}+\frac{\partial \dot{x}}{\partial a}a\Delta t+O\left(3\right) \end{array}$$ (14)

where Δ*t* = *t* − *t*_0_ and *O*(3) represents terms of order 3 and above. Fig 10 shows a state, **x**_1_, perturbed forward in time and another, **x**_2_, perturbed in space and time. Representing the perturbed states **x**_1_ and **x**_2_ as their Taylor expansions and substituting $\dot{x}=f$ we obtain

$$\begin{array}{ccc} x_{1} & = & x_{0}+f\Delta t+\frac{1}{2}\dot{f}\Delta t^{2}+O\left(3\right)\\ x_{2} & = & x_{0}+f\Delta t+\frac{\partial x}{\partial a}a+\frac{1}{2}\dot{f}\Delta t^{2}+\frac{1}{2}\frac{\partial^{2}x}{\partial a^{2}}a^{2}+\frac{\partial f}{\partial a}a\Delta t+O\left(3\right) \end{array}$$ (15)

where **f** = **f**(**x**_0_, *t*_0_). Now, from Eq (15) and recognising that $\frac{\partial x}{\partial a}=v$, the rate of stretch is readily obtained as

$$\begin{array}{c} \lim_{\begin{array}{c} a\to 0\\ \Delta t\to 0 \end{array}}\frac{(x_{2}-x_{1})-av}{a\Delta t}=\lim_{\begin{array}{c} a\to 0\\ \Delta t\to 0 \end{array}}\left(\frac{\partial f}{\partial a}+\frac{O(3)}{a\Delta t}\right)=\frac{\partial f}{\partial a} \end{array}$$ (16)

It is easily seen that

$$\begin{array}{c} \frac{\partial f}{\partial a}=Jv \end{array}$$ (17)

Where **J** is the Jacobi matrix. If **v** is an eigenvector with a real eigenvalue, the local stretch is parallel to it. If it is complex, the stretch will lie in the space spanned by **v** and its complex conjugate (since the flow is real). Thus, imagnining for a moment a full spectral decomposition, it is easily seen that, locally, the only directions of positive stretch are those associated with eigenvalues with a positive real part. Nearby solutions will converge exponentially in all other directions—i.e. contract. This holds for either autonomous or non-autonomous systems as no assumptions have been made in regards to the time dependence of $\dot{x}\left(x,t\right)$ apart from the requirement of smoothness. So, as is evident, the spectrum plays a pivotal role in manifold orientation, even for non-autonomous systems.

Although the above is not a novel result, its implications justify its repetition here. Chaos is a global phenomenon and eigenvalue decompositions cannot be utilised globally to describe stretching and folding. However, *locally* and *instantaneously*, stretching and folding are described by the spectrum of the (ever changing) Jacobi matrix and the recognition of this underpins the present method.

#### Spatial discretisation

The choice of spatial discretisation method is not central to the method, and the approach taken here may well be substituted with another method depending on application. Here, all fields on the BM are discretised using finite element theory with Hermite shape functions:

$$\begin{array}{ccc} & c\left(m,t\right)=\sum_{i=1}^{K}\phi_{i}\left(m\right)c_{i}\left(t\right) & \dot{c}\left(m,t\right)=\sum_{i=1}^{K}\phi_{i}\left(m\right){\dot{c}}_{i}\left(t\right)\\ & u_{j}\left(m,t\right)=\sum_{i=1}^{K}\phi_{i}\left(m\right)u_{ji}\left(t\right) & w_{j}\left(m,t\right)=\sum_{i=1}^{K}\phi_{i}\left(m\right)w_{ji}\left(t\right)\\ & d_{j}\left(m,t\right)=\sum_{i=1}^{K}\phi_{i}\left(m\right)d_{ji}\left(t\right),\;j=1,2,...M \end{array}$$ (18)

where *ϕ*_*i*_(*m*) are the shape functions, K is the number of degrees of freedom used to discretise the BM and, e.g.,

$$\begin{array}{c} c_{i}=c_{ij},\;i=1,2,...K,\;j=1,2,...N \end{array}$$ (19)

are nodal values of the position of the BM and *N* is the dimension of the system. Time derivatives and spatial derivatives for the BM are easily obtained from these expressions. The use of Hermite (higher order) shape functions allows for the definition of curvature, which appears in Eq (6). All simulations use the same 40-node (*K* = 80) mesh of 1-D elements with isoparametric Hermite shape functions. Numerical integration over *m* is performed with a full Gauss integration scheme with 7 Gauss points per element (*K*_*G*_ = 280). Nodal updates are determined by solving for nodal rates and integrating numerically over *t* with an explicit time integration scheme. The forward Euler method is used for ease of implementation.

Introducing the approximations in Eq (18), multiplying Eq (3) by *ϕ*_*j*_ and integrating over *m* on both sides, we obtain

$$\begin{array}{c} \oint_{m}\sum_{i}\phi_{i}{\dot{c}}_{i}\phi_{j}dm=\oint_{m}\left(f-u_{1}\left(f\cdot u_{1}\right)-u_{2}\left(f\cdot u_{2}\right)+u_{1}d_{1}+u_{2}d_{2}\right)\phi_{j}dm,\;j=1,2,..,K \end{array}$$ (20)

Which can be written in matrix form and solved as

$$\begin{array}{c} {\dot{c}}_{i}=\sum_{j=1}^{K}B_{ij}r_{j},\;B=A^{-1},\;i=1,2,...K \end{array}$$ (21)

where

$$\begin{array}{ccc} r_{j} & = & \oint_{m}\left(f-u_{1}\left(f\cdot u_{1}\right)-u_{2}\left(f\cdot u_{2}\right)+u_{1}d_{1}+u_{2}d_{2}\right)\phi_{j}dm\\ A & = & A_{ij}=\oint_{m}\phi_{i}\phi_{j}dm \end{array}$$ (22)

Now, the time integration scheme can be utilised to update **c**. The system matrices from the finite element formulation do not change between time steps, which can be exploited for efficiency.

For a higher order attractor, the expression for the right hand side is modified according to the attractor dimension *M* as

$$\begin{array}{c} r_{j}=\oint_{m}\left(f-\sum_{i=1}^{M}u_{i}\left(f\cdot u_{i}\right)+\sum_{i=1}^{M}u_{i}d_{i}\right)\phi_{j}dm \end{array}$$ (23)

The inward velocity *d*_1_ can be evaluated at each point on **c** directly using Eq (6). The tangential velocity *d*_2_ is found by the finite element solution to Eq (9):

$$\begin{array}{c} d_{2i}=\frac{1}{\beta_{2}}\sum_{j=1}^{K}B_{ij}q_{j},\;i=1,2,...K \end{array}$$ (24)

where

$$\begin{array}{c} q_{j}=\oint_{m}\left(\left(c^{\prime }\cdot u_{2}\right)u_{2}\cdot \left(u_{2}\phi_{j}^{\prime }+u_{2}^{\prime }\phi_{j}\right)\right)dm \end{array}$$ (25)

#### Auxiliary manifold orientation - concepts

There exists an extensive body of literature on how to define and practically determine invariant manifolds. A comprehensive overview is given by Gorbana and co-workers in [53]. This is an active area of research and new highly advanced methods are continually being developed [54]. Here, an approach is taken, which is somewhat related to what is labelled the “method of invariant grids” in [53]. It differs substantially in that the tangent field of the invariant manifold is *not* found by numerical differentiation in phase space. This approach, though intuitive, requires refined meshes and is prone to numerical instability. Instead, a hybrid approach is taken, where AM orientation is determined by a spectral method, but *selection* of eigenvectors from this spectrum, and thus orientation, makes use of the grid. The reasoning behind this choice is pragmatic and based on experience: Even if very large time steps are taken between two orientation updates, the tangential motion of the AM in between these updates will be small when compared to typical mesh refinement. Furthermore, the use of a spectral method renders the orientation of the inward normal **u**_1_ independent of interior points. The obvious drawback is the need for solution of the eigenvalue problem at each Gauss point and at every time step. A redeeming feature is that this can be done efficiently by use of sparse iterative solvers that solve for only the *M* necessary eigenvalues and utilise eigenvalues from previous time steps as initial guesses. The current software implementation does not exploit these features and computes the entire spectrum from full matrices using direct methods.

Locally, the tangent space of the AM is spanned by the tangent vectors $w_{i}\in R^{N}$ where *i* = 1, 2, …, *M*. Chaotic motion is often described as successive processes of stretching and folding. As discussed, locally, these processes correspond to exponential growth and decay, respectively. The selection of the tangent vectors **w**_*i*_ is such that every direction in which stretch occurs is contained within the AM. In this way, every off-manifold motion will be an exponential decay onto the manifold.

Consider the Jacobi matrix of Eq (2)

$$\begin{array}{c} J=\partial \dot{x}/\partial x \end{array}$$ (26)

In any non-linear system, the Jacobi matrix depends on the state, i.e., **J** = **J**(**x**). In non-linear non-autonomous systems it may also depend explicitly on time, i.e. **J** = **J**(**x**, *t*). The method is equally applicable in both of these situations.

The AM is oriented such that all directions of stretch are contained within it at all times. Locally and instantaneously, these directions are those spanned by eigenvectors associated with eigenvalues with a positive real part. That is, the manifold tangent vectors **w**_*i*_ are selected such that they span the same space as solutions to the local system associated with directions of stretch. The number of eigenvalues with positive real parts determines the required dimension of the AM. For two positive real parts at least a surface is required, and for three a 3-D hypersurface, and so on. Knowing the dimension of the attractor a priori is advisable, since then a good (if not perfect) indication of the dimension of the AM is then known. A good account of the estimation of attractor dimension, for both experimental data and systems with known ODEs, is given by Wolf and co-workers [55].

When a direction of stretch with associated eigenvalue *λ* is encountered somewhere in phase space, there are two situations

1. λ∈C: The directions of stretch lie on a surface spanned locally by $v_{1}e^{\lambda t}+v_{2}e^{\bar{\lambda }t},\;\forall t$, where **v**_1_ and **v**_2_ are the eigenvectors associated with eigenvalues *λ* and $\bar{\lambda }$.
2. λ∈R: The direction of stretch is parallel with the eigenvector associated with *λ*.

**Fig 11:**
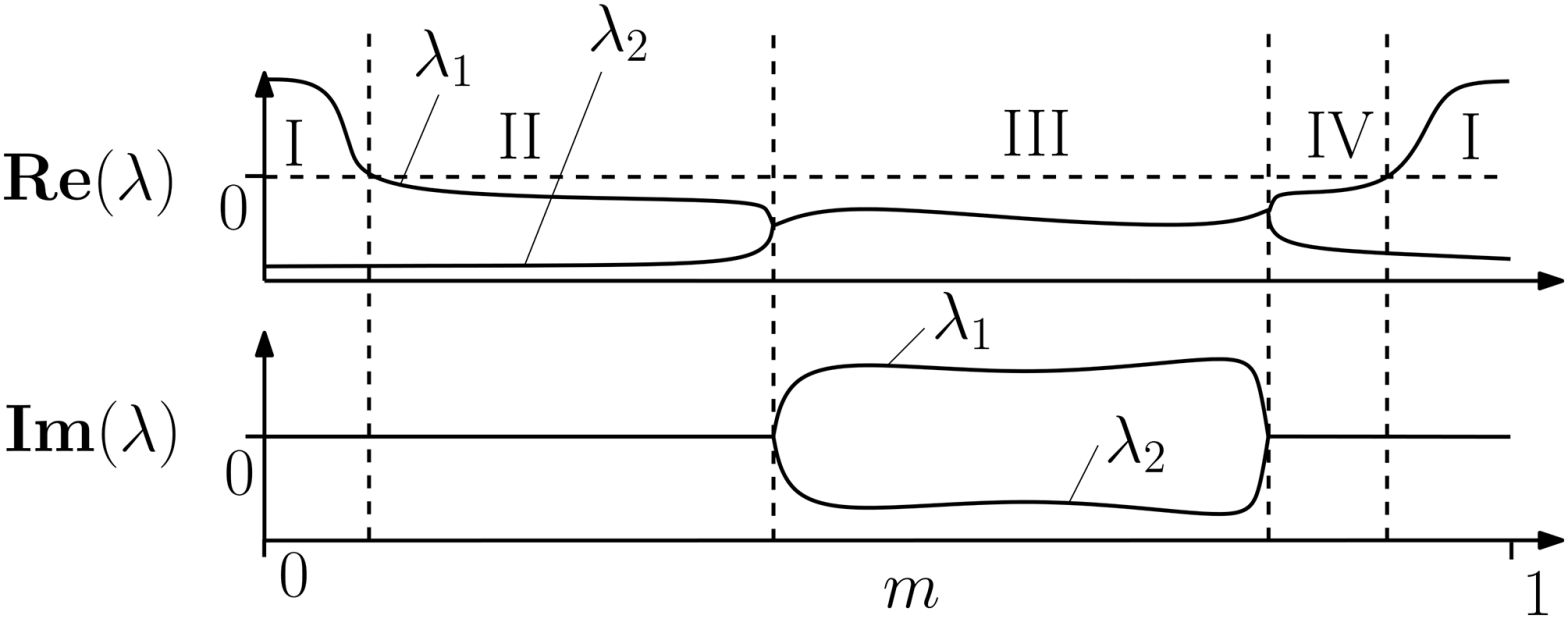
Schematic showing the evolution of two key eigenvalues along the entire length of the BM, i.e, for *m* ∈ [0, 1[. Note that *λ*_1_ has a positive real part in sub-domain I, which is negative in sub-domain II, III and IV. In sub-domain III *λ*_1_ is complex with the complex conjugate *λ*_2_. Due to the requirement of continuity, the local solution space associated with *λ*_1_ and *λ*_2_ must be spanned by **v**_1_ and **v**_2_ along the entire BM. Thus, the fact that one eigenvalue has a positive real part for only a portion of the BM, means that the motion corresponding to two eigenvalues must be spanned along the entire BM.

In case 1, one may define the local tangent vectors in any way that span the same space as $v_{1}e^{\lambda t}+v_{2}e^{\bar{\lambda }t}$, e.g., by selecting two distinct phases: 0 (giving **v**_1_ + **v**_2_) and +/−*i* (giving *i* **v**_1_ − *i* **v**_2_). If the flow is real **v**_1_ and **v**_2_ appear as complex conjugate, so the space is spanned by Re(**v**_1_) and Im(**v**_1_). These vectors are then Gram-Schmidt orthonormalised to yield the orthonormal basis (**w**_1_,**w**_2_) of the AM. With the requirement of smoothness, the orientation of the AM is defined even in regions of phase space with no directions of stretch. This implies the requirement that **J**(**x**) is continuous and differentiable. Even if a direction of stretch is associated with a positive real eigenvalue, there may be another region of phase space where this eigenvalue becomes complex (with positive or negative real part) and thus requires a second direction of expansion. The only way to know this is to trace the eigenvalue along the AM and track its evolution. A schematic illustrating this process is shown in Fig 11. The phenomenon of directions of stretch varying in number across phase space is well known and is what is labelled “unstable dimension variability” in, e.g., [39].

#### Auxiliary manifold orientation—initialisation

Selecting the appropriate eigenvectors during initialisation takes a two step approach: First, the eigenvalues of all Gauss points on the BM are determined until a node is reached where at least one eigenvalue has a positive real part. Subsequently, the rest of the Gauss points are scanned incrementally along the BM selecting the ones corresponding with minimum increments in modulus. If the discretisation of the BM and AM is sufficiently fine, the minimum increment selection criterion will correspond to continuity. Thus, eigenvalues for neighbouring points on the AM and the BM can be directly organised and numbered consistently. Although it is somewhat computationally wasteful, it is conceptually more straight forward to perform the sorting process before the selection of eigenvalues. This is illustrated as pseudo-code in algorithm 1.

**Algorithm 1:** Sort eigenvalues for point-to-point continuity

*m* ← *m*_1_

**J** ← **J**(**c**(*m*), *t*_0_)

Solve $Jv_{j}^{\left(1\right)}=\lambda_{j}^{\left(1\right)}v_{j}^{\left(1\right)}$ for $\lambda_{j}^{\left(1\right)}$ and $v_{j}^{\left(1\right)},\;j=1,2,..N$

Loop over Gauss points

**for** *p* = 2, 3, …, *K*_*G*_ **do**

 *m* ← *m*_*p*_

 **J** ← **J**(**c**(*m*), *t*_0_)

 Solve **J** **v**_*i*_ = *λ*_*i*_ **v**_*i*_ for *λ*_*i*_ and **v**_*i*_, *i* = 1, 2, …*N*

 Now sort eigenvalues applying principle of continuity

 **for** *j* = 1, 2, …, *N* **do**

  Select *λ*_*i*_ from spectrum such that

$|\lambda_{j}^{\left(p-1\right)}-\lambda_{i}|$ is minimised

$\lambda_{j}^{\left(p\right)}\leftarrow \lambda_{i}$

 **end**

**end**

With the eigenvalues sorted, the local orientation vectors are easily identified as those eigenvectors, **v**_*j*_, where $\text{Re}(\lambda_{j}^{\left(p\right)})>0$ at any point along the BM (*m*_*p*_, *p* = 1, 2, …, *K*_*G*_). Let them be identified as 1s in a binary array *l*_*j*_. The principles of the identification procedure is shown as pseudocode in algorithm 2.

**Algorithm 2:** Identify directions of stretch

*l*_*j*_ ← 0, *j* = 1, 2, … *N*

**for** *p* = 1, …, *K*_*G*_ **do**

 **for** *j* = 1, 2, …, *N* **do**

  **if** $\text{Re}(\lambda_{j}^{\left(p\right)}>0)>0$ **then**

   *l*_*j*_ ← 1 **end**

  **end**

 **end**

**end**

Now, the number of entries in *l*_*j*_ equalling 1, should exceed the a-priori predicted dimension of the attractor. If it does not, then the initialisation of the BM is inadequate and another must be tried.

The above pseudocode is written to communicate the concept of the method and is not optimised. For example, there is no need to sort eigenvalues that do not correspond to directions of stretch.

#### Auxiliary manifold orientation—updates

Updating $\lambda_{j}^{\left(p\right)}$ at each time step simply follows the principle of continuity in time, so updates in eigenvalues are those minimizing changes in modulus from the previous time step. At each time step, the inward normal **u**_1_ is projected onto the orthonormal basis associated with the corresponding eigenvectors. Thus, at each time step, Gauss point and interior point the Jacobi matrix is updated, the eigenvalues of the Jacobi matrix are calculated, and the eigenvectors associated with minimum change in eigenvalue modulus are used to update the orientation of the manifold. If Δ*t*_*λ*_ is sufficiently small, this corresponds to continuity in time of manifold orientation. The process of time integration and manifold updates is outlined as pseudo-code in algorithm 3. Note that the time step between orientation updates is larger than the time step for position updates. For clarity, the algorithm is written for a 2-D AM (*M* = 2) with $\lambda_{1}^{\left(p\right)}$ and $\lambda_{2}^{\left(p\right)}$ corresponding to the eigenvalues of orientation at gauss point *m*_*p*_. Generalisation to higher dimension is straight forward.

**Algorithm 3:** Time integration

Initialisation. **while** *t* < *T* **do**

 *i* ← *i*+1

 *t*_*i*_ ← *t*_*i*−1_+Δ*t*

 t* ← t*+Δ*t*

 solve Eq (21) for $\dot{c}$

$c\leftarrow c+\Delta t\dot{c}$

 **if** t* > Δ*t*_*λ*_ **then**

  t* ← 0

  Loop over gauss points on the BM:

  **for** *p* = 1, 2, …, *K*_*G*_ **do**

   *m* ← *m*_*p*_

   **J** = **J**(**c**(*m*), *t*_*i*_)

   Solve **J** **v**_*j*_ = *λ*_*j*_ **v**_*j*_ for *λ*_*j*_ and **v**_*j*_, *j* = 1, 2, …*N*

   Select *λ*_*j*_ from the spectrum of eigenvalues such that

$|\lambda_{1}^{\left(p\right)}-\lambda_{j}|$ is minimised

$\lambda_{1}^{\left(p\right)}\leftarrow \lambda_{j}$

   Select *λ*_*k*_ from the spectrum of eigenvalues such that

$|\lambda_{2}^{\left(p\right)}-\lambda_{k}|$ is minimised (s.t. *k* ≠ *j*)

$\lambda_{2}^{\left(p\right)}\leftarrow \lambda_{k}$

   **If** $\lambda_{1}^{\left(p\right)}$ is complex (*λ*_2_ is conjugate) **then**

$w_{1}^{*}\leftarrow \text{Re}\left(v_{j}\right)$

$w_{2}^{*}\leftarrow \text{Im}\left(v_{j}\right)$

   **else**

$w_{1}^{*}\leftarrow v_{j}$

$w_{2}^{*}\leftarrow v_{k}$

   **end**

   Gram-Schmidt orthonormalise $\left(w_{1}^{*},w_{2}^{*}\right)$ to yield (**w**_1_ **w**_2_).

   Update inward normal by projecting onto tangent space of AM:

$u_{1}^{(p)*}\leftarrow \left(u_{1}^{\left(p\right)}\cdot w_{1}\right)w_{1}+\left(u_{1}^{\left(p\right)}\cdot w_{2}\right)w_{2}$

   All other directions (in this case one) appear as tangent space of the BM:

$u_{2}^{\left(p\right)}\leftarrow \frac{c^{\prime }\left(m\right)}{|c^{\prime }\left(m\right)|}$

   Gram-Schmidt orthonormalise $u_{1}^{(p)*}$ with respect to $u_{2}^{\left(p\right)}$ (and other tangent vectors if higher-D) to yield $u_{1}^{\left(p\right)}$

  **end**

 **end**

**end**

At the first time step, updates in orientation are associated with large changes in **u**_1_ if the initial guess is poor. Conversely, if the initial guess for the AM is close to the real, long term, AM, the projection will not alter **u**_1_ much. In this paper, a flat, circular surface is used as an initial guess and initial values of **w**_1_ and **w**_2_ computed on this surface. This approach works well when off-manifold directions are almost in phase with on-manifold location, but may fail if off-manifold dynamics are out of phase, potentially causing the AM to become degenerate and numerically unstable. Thus, convergence depends on a reasonable initial guess. This which might potentially be generated from Poincaré sections in future incarnations of the method. Fig 3. shows a manifold changing its shape in the initial transient phase of a simulation.

**Fig 12:**
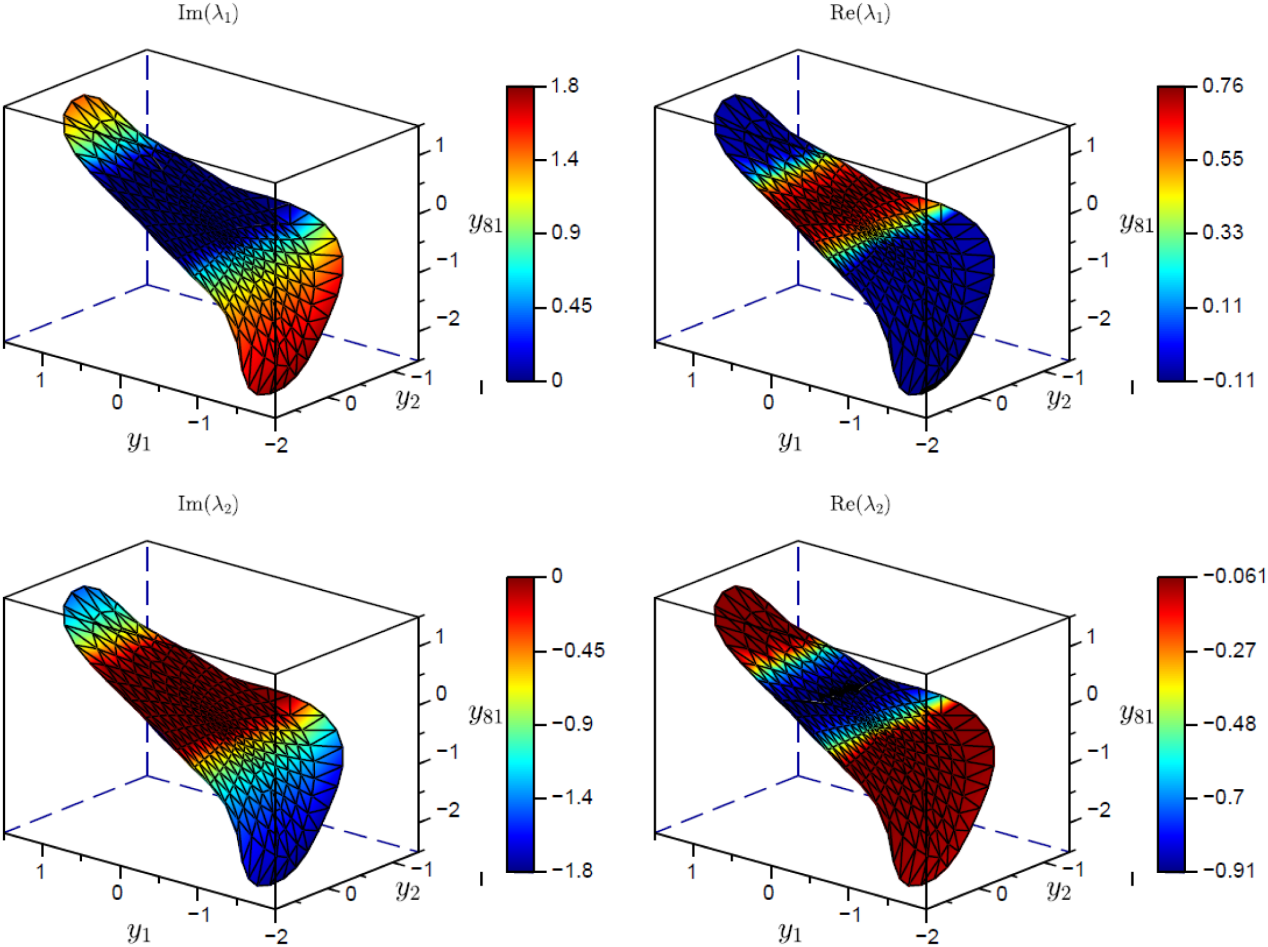
Eigenvalues associated with AM eigenvectors at *tω*/(2*π*) = 4.75 for Eq (12). The parameters (set III) are given in Table 1.

Fig 12 depicts the converged manifold and the manifold eigenvalues for parameter set III in Table 1. The manifold is projected to the (*y*_1_, *y*_2_, *y*_81_) space. Where complex, as expected, the two eigenvalues of orientation are complex conjugate. There is a central region of stretch where *λ*_1_ is positive. Conceptually, this corresponds to the area around the unstable saddle equilibrium at (0, 0) in the unmodified 2-D Duffing system.

## Discussion

A practical method for calculating extrema of chaotic systems has been presented here. The equations of the method are written such that they can be wrapped around an underlying system of which the detailed structure is less important, as long as its flow is continuous and differentiable. The simulation results are post-transient extrema of systems with fairly fast, close to in-phase, off-manifold dynamics. Such systems pose the least challenging in terms of defining (guessing) an initial shape, that will rapidly converge onto the real AM. Addressing this challenge, for example by use of fits to Poincaré sections, would increase the chances of convergence.

The presented method has drawbacks which should be addressed in future work: Firstly, it will not distinguish between basins of attraction as the BM is defined a priori as a single closed curve. For this, an approach based on the level set method would be superior but perhaps at the cost of added computational work. Secondly, the presence of noise would also make the BM itself noisy. The method in its current incarnation offers no strategies to deal with this added challenge, which will inevitably come with the application to experimental data. Thirdly, the adaptation to experimental data would entail a phase space reconstruction step. The method, in its current incarnation, requires knowledge of the rates of all phase space variables, so will need to be modified if phase space reconstruction techniques based on time-delay coordinates [56, 57] are to be employed. However, it may well be that a hybrid scheme where some rates are inferred from knowledge of the rest would suffice.

The computational benefits associated with defining the BM as low-D as possible are potentially enormous. Consider the cost of operating on a conventional *N* − 1 BM rather than an *M* − 1 ditto. The memory needed to store the BM, let alone operate on it, scales as *K*^*D*^, where *D* is BM dimension and *K* is the resolution of the BM, e.g., as in this case, the number of nodes of a finite element formulation. Plugging in the numbers for even modest sized systems quickly reveals why the *D* = *N* − 1 approach is infeasible. For example, storing an *N* − 1 BM, for an *N* = 82 phase space system with identical resolution to the one used in the presented analyses requires 1.13 ⋅ 10^155^ bytes if stored as double precision.

## Conclusion

Based on the presented results, it is concluded that it is practically possible to predict extrema for large differentiable chaotic systems and that these extrema have the potential to display harmonic behaviour. This enables the efficient calculation of extrema of chaotic systems in the long term once the post-transient regime is reached.

## Supporting information

S1 File Bounding manifold and interior points. Projection of the BM, interior points and spacing truss elements into (*y*_1_, *y*_2_, *y*_81_) space for 5 forcing cycles. Overlaid are 20 solutions to the ODE. The manifold and solutions are seen to converge.
(AVI)

S2 File Bounding manifold. Projection of BM onto coordinate surfaces (*y*_1_, *y*_2_), …, (*y*_81_, *y*_82_) for 5 forcing cycles of parameter set III.
(AVI)
